# Social disparities in the prevalence of diabetes in Australia and in the development of end stage renal disease due to diabetes for Aboriginal and Torres Strait Islanders in Australia and Maori and Pacific Islanders in New Zealand

**DOI:** 10.1186/s12889-017-4807-5

**Published:** 2017-10-11

**Authors:** Kathleen Hill, Paul Ward, Blair S. Grace, Jonathan Gleadle

**Affiliations:** 1 Discipline of Public Health, Flinders University, Adelaide, South Australia Australia; 2 Faculty of Health and Medical Sciences, University of Adelaide, Adelaide, South Australia Australia; 3 School of Medicine, Flinders University, Adelaide, South Australia Australia

**Keywords:** Diabetes, Socioeconomic status, Aboriginal and Torres Strait islanders, Renal disease, Australia, New Zealand

## Abstract

**Background:**

Disparities in health status occur between people with differing socioeconomic status and disadvantaged groups usually have the highest risk exposure and the worst health outcome. We sought to examine the social disparities in the population prevalence of diabetes and in the development of treated end stage renal disease due to type 1 diabetes which has not previously been studied in Australia and New Zealand in isolation from type 2 diabetes.

**Methods:**

This observational study examined the population prevalence of diabetes in a sample of the Australian population (7,434,492) using data from the National Diabetes Services Scheme and of treated end stage renal disease due to diabetes using data from the Australian and New Zealand Dialysis and Transplant Registry. The data were then correlated with the Australian Bureau of Statistics Socioeconomic Indexes for Areas for an examination of socioeconomic disparities.

**Results:**

There is a social gradient in the prevalence of diabetes in Australia with disease incidence decreasing incrementally with increasing affluence (Spearman’s rho = .765 *p* < 0.001). There is a higher risk of developing end stage renal disease due to type 1 diabetes for males with low socioeconomic status (RR 1.20; CI 1.002–1.459) in comparison to females with low socioeconomic status. In Australia and New Zealand Aboriginal and Torres Strait Islanders, Maori and Pacific Islanders appear to have a low risk of end stage renal disease due to type 1 diabetes but continue to carry a vastly disproportionate burden of end stage renal disease due to type 2 diabetes (RR 6.57 CI 6.04–7.14 & 6.48 CI 6.02–6.97 respectively *p* < 0.001) in comparison to other Australian and New Zealanders.

**Conclusion:**

Whilst low socioeconomic status is associated with a higher prevalence of diabetes the inverse social gradient seen in this study has not previously been reported. The social disparity seen in relation to treated end stage renal disease due to type 2 diabetes for Aboriginal and Torres Strait Islanders, Maori and Pacific Islanders has changed very little in the past 20 years. Addressing the increasing incidence of diabetes in Australia requires consideration of the underlying social determinants of health.

## Background

Over one million Australians are living with diabetes and 10% of these have type 1 diabetes mellitus (T1DM). The childhood incidence rate of T1DM in Australia (22.5 per 100,000 population) is one of the highest in the world [1] and sits in the top 10 of countries compared across the globe [2]. The population prevalence of type 2 diabetes mellitus (T2DM) in Australia is similar to European countries at 5%, however a higher incidence is seen in New Zealand (7.3%) and has also been demonstrated to be much higher in other countries, for example in Malaysia (17.9%) [3]. Whilst the onset of T2DM is largely attributed to age and obesity with some evidence of predisposing genetic risk [4], the aetiology of T1DM remains unknown. The process through which pancreatic beta cell destruction occurs is autoimmune however it is thought to occur in genetically susceptible individuals and a wide number of hypotheses regarding an environmental trigger have been studied but not yet proven [5]. In Australia a higher prevalence of T2DM is known to be associated with low area socioeconomic status (SES) [6] and potentially related to higher rates of obesity. In contrast to T2DM no association with low area SES has previously been demonstrated for T1DM [7–9]. In Europe, Canada and the United States socioeconomically disadvantaged people have higher rates of morbidity and mortality in T1DM [10–14]. However very little is currently known regarding T1DM prevalence in Australia and the disease outcome of treated end stage renal disease (ESRD) due to T1DM for people with low area SES, this regions Indigenous minority populations, Aboriginal and Torres Strait Islanders, and Maori and Pacific Islanders. This study is an exploration of social disparities in diabetes in relation to area SES and Aboriginal and Torres Strait Islander, Maori and Pacific Islander ethnicity.

In 2014, there were 25,626 people in Australia and New Zealand receiving renal replacement therapy for ESRD, and 37% of new cases (*n* = 954) were attributed to diabetic nephropathy [15]. In this region, people with low area SES are more likely to progress to ESRD due to kidney disease of any aetiology, but this disparity is most pronounced in diabetic nephropathy caused by T2DM [16, 17] with a relative risk of progression to ESRD for the most disadvantaged decile versus the most advantaged decile of 2.38 [17]. While studies from other countries have found a higher likelihood of ESRD due to T1DM for people with low SES [18, 19] this has not previously been studied separately from T2DM in Australia. The incidence of T1DM in the Aboriginal and Torres Strait Islander population is reported to be 9 per 100,000 but this may be an underestimate because of lower levels of registration with the National Diabetes Services Scheme and underreporting of Indigenous status at diagnosis [7]. Although the incidence of T1DM nephropathy has not previously been reported for Aboriginal and Torres Strait Islanders, Maori and Pacific Islanders, the incidence of progression to ESRD from chronic kidney disease in these populations due to any cause is much higher than the general population [20]. It is also known that Aboriginal and Torres Strait Islanders, Maori and Pacific Islanders have a disproportionate burden of ESRD [15] from diabetic nephropathy, which is attributed to a higher prevalence of T2DM but also to an increased likelihood of progressive renal disease [20]. This has largely been attributed to individual behaviours [20] however limited access to appropriate health care services is a strong determinant of health outcomes [21].

## Methods

### Aims

The aims of this study were to examine the population prevalence of diabetes in Australia for an association with area SES. This study also sought to determine whether there is any relationship between T1DM ESRD and area SES in Australia, and to re-examine diabetic nephropathy ESRD for this region’s most socially disadvantaged and vulnerable minority populations: Aboriginal and Torres Strait Islanders in Australia, and Maori and Pacific Islanders in New Zealand.

### Study design

This observational study is a secondary analysis of existing data using an ecological design to make large scale comparisons between groups [22]. Ethical approval for the research was obtained from the Southern Adelaide Clinical Human Research Ethics Committee (SACHREC), reference number 564.13.

### Study period

This study examined all reported cases of T1DM ESRD for a 5 year period from 2008 to 2012 and the Australian population prevalence of diabetes as recorded in 2014.

### Data sources

#### Treated end stage renal disease

The Australia and New Zealand Dialysis and Transplant Registry (ANZDATA), located within the South Australian Health and Medical Research Institute, record the incidence and outcomes of dialysis and transplant treatment for people with ESRD and receive their data annually from individual Renal units. The information collected by the ANZDATA Registry is used for research that is of benefit to the data contributors, a process overseen by a network of nephrologists, surgeons and renal nurses with interest and expertise in using the data [23]. Australian population data and individual-level de-identified data (gender, age, age of development of ESRD) for all cases of ESRD due to T1DM for a five-year period (*n* = 534) were obtained from the ANZDATA Registry. Data were also obtained on the relative risk ratio (RR) for T1DM and T2DM ESRD for Aboriginal and Torres Strait Islanders and Maori and Pacific Islanders through an analysis of all data held in the registry.

### Socioeconomic status

The Socio-Economic Indexes for Areas (SEIFA) is an index developed by the Australian Bureau of Statistics that ranks areas in Australia according to relative socioeconomic advantage based on information from the five-yearly Census [24]. The structure of SEIFA allows measurement of key components of area SES such as income, wealth, social class, occupation, education and community cohesion and individual data from within a post code area is aggregated to rank that post code by area SES. A SEIFA index summarises the characteristics of people and households within an Australian postal area. The combination of these measures allows an integration of the individual, household and community factors that can influence health [25]. Data in SEIFA are ranked in deciles from 1 (lowest area SES) to 10 (highest area SES). ANZDATA records the residential postcode for each Australian case of ESRD and these post codes were used to explore population prevalence of diabetes to estimate the relative risk of ESRD. The postal areas SEIFA (2011) was used to identify the area SES for each postcode. The population in each post code ranged from 294 to 102,224 however the median population per post code was 17,467 people.

### The prevalence of diabetes in a sample of the Australian population

This study examined the population prevalence of diabetes in a cross sectional way rather than a whole of region way using the T1DM ESRD post codes as a sample. Using the post codes for each case of T1DM ESRD the total size of the population studied for diabetes prevalence was 7,434,492 which represents one third of the Australian population. Population size (number of people) in each postcode with a case of T1DM ESRD was recorded, and the population prevalence of diabetes in that postcode (number of people with diabetes) which is recorded by the National Diabetes Service Scheme registrant database was then used to calculate diabetes prevalence as a percentage. The data were then converted into population prevalence by SEIFA decile using the Australian Bureau of Statistics database which lists the SEIFA decile for each postcode. People newly diagnosed with diabetes register with the National Diabetes Services Scheme and the register is thought to have almost complete coverage.

### Statistical analysis

The characteristics of the data determined the descriptive and inferential statistics used through an examination of normality, distribution and the level of measurement [26]. For the continuous data, Levine’s tests of normality were used, which have an assumption that the variance between groups is normally distributed. For normally distributed data, the parametric t-test was used for independent variables with two categories, and an analysis of variance (ANOVA) was used for independent variables with more than two categories. Where the continuous dependent variable was not normally distributed and the independent variable had more than two categories, the non-parametric Kruskal–Wallis tests and Spearman’s rank correlation coefficients were used [27, 28]. To calculate the relative risk of T1DM ESRD in relation to area SES the 10 SEIFA deciles were further condensed into two categories of low area SES (deciles 1–5) and high area SES (deciles 6–10). Relative risk estimates were calculated within a chi-squared analysis. Data were analysed in SPSS Statistics for Windows, version 21, IBM Corp., Armonk, NY; IBM Corp 2012.

## Results

### Prevalence of diabetes mellitus in the population sample (2014)

**Fig. 1:**
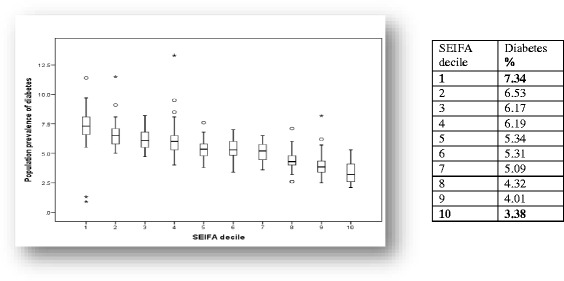
Population prevalence (percentage) of any form of diabetes by collation of the sample post codes ranked into SEIFA deciles from 1 (lowest area SES) to 10 (highest area SES) 2014

Population prevalence for any form of diabetes was plotted against the SEIFA deciles.^1^ The mean prevalence of diabetes was 5.3% (IQR 4.2–6.3, SD 1.63). The prevalence of diabetes in the Australian population varies considerably by SEIFA decile. There was an incremental increase in prevalence as area SES decreased, with the lowest SEIFA decile having a prevalence of diabetes more than double that of the highest decile, demonstrating an inverse social gradient (Fig. 1). The correlation coefficient for association between SEIFA decile and the percentage of the population with diabetes was −.765 (Spearman’s *p* < 0.001).

**Fig. 2:**
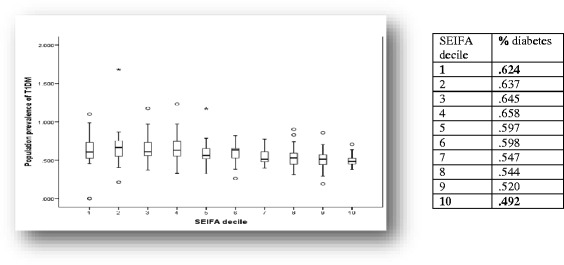
Population prevalence (percentage) of T1DM in Australia by collation of the sample post codes ranked into SEIFA deciles from 1 (lowest area SES) to 10 (highest area SES) 2014

Most diabetes cases in Fig. 1 are T2DM (85%) with the remaining 15% comprising of T1DM (12%) and gestational diabetes (3%). The outliers seen in Figs. 1 and 2 were included in the analysis. They represent postcodes (communities) with a much higher or much lower prevalence than that seen in the decile overall and may represent familial clustering or a particularly high density of a single minority ethnic group, both of which are known to be associated with diabetes prevalence.

### Prevalence of type 1 diabetes mellitus in the population sample (2014)

In Fig. 2, T1DM data are presented separately to all other types of diabetes. The mean prevalence of T1DM was 0.588% of the population (IQR 0.49–0.66, SD 0.161). The national prevalence of T1DM is known to be 0.5% of the population and previously this has been reported as not being associated with area SES [29]. The results show that population prevalence of T1DM in this study followed a small but statistically significant inverse gradient with higher area SES deciles having an overall lower population prevalence of T1DM (Fig. 2).^2^ The correlation coefficient for association between SEIFA decile and the percentage of the population with T1DM was −.397 (Spearman’s *p* < 0.001).

### Type 1 diabetes mellitus treated end stage renal disease

Incidence rates (new presentations) of T1DM ESRD in Australia are shown in Table 1. Rates have been steadily increasing but there was a much lower incidence for people of Aboriginal and Torres Strait Islander ethnicity. The crude incidence of ESRD due to any cause per million population was 112 [15] with T1DM representing about 4.9% of cases in 2012.

**Table 1:** T1DM ESRD incidence rates per million population with the lower and upper bound values for the period 2008–2012

Year	Australia (non-Aboriginal and Torres Strait Islander)	Aboriginal and Torres Strait Islander (Australia)	Maori (NZ)
2008	5.90 (4.69–7.34)	0.26 (0.03–0.95)	6.22 (1.69–15.93)
2009	4.86 (3.77–6.16)	0.51 (0.14–1.33)	6.12 (1.66–15.68)
2010	5.43 (4.29–6.79)	0.63 (0.20–1.48)	3.01 (0.36–10.88)
2011	6.50 (5.25–7.96)	0.12 (0.003–0.69)	5.93 (1.61–15.20)
2012	8.27 (6.85–9.89)	0.36 (0.07–1.06)	^a^n/a

^a^no Maori cases reported for this year

### Age of presentation with type 1 diabetes mellitus and end stage renal disease

There was an even distribution of age of onset for incident T1DM ESRD cases for both males (*n* = 303) and females (*n* = 231). Table 2 describes the incidence rates of T1DM ESRD during the period studied by age group. The youngest person diagnosed with T1DM ESRD in the period was aged 14 years and the oldest was 81 years. The mean age of presentation was 45 (SD 11.72) and very few cases were diagnosed after the age of 60 years.

**Table 2:** T1DM ESRD new cases for the period 2008–2012 classified by age group

Age group	Percentage of new cases
< 20	0.2%
21–30	10.8%
31–40	29%
41–50	30%
51–60	21%
> 60	9%

Age of onset of ESRD in T1DM did not differ significantly by gender (*p* = .612) however more males developed T1DM ESRD than females (56.3% versus 43.3%). The extremely small number of Aboriginal and Torres Strait Islanders and Maori seen over this period make a comparison on age of onset between groups unreliable.

### Type 1 diabetes mellitus treated end stage renal disease, socioeconomic status, age and gender

There were similar numbers of incident cases of T1DM ESRD in each SEIFA decile from the lowest area SES (1) through to 9 (range 43–50) with a slightly smaller number (32) in decile 10, the most advantaged area SES. There was little difference in age of presentation by area SES, with the mean age being almost identical between deciles 1 (47.31) and 10 (47.19). A multivariate one-way ANOVA demonstrated no significant difference between the age of presentation in relation to area SES for males or females (*p =* .766, *p* = .289). However, males of low area SES were overall at increased risk of T1DM ESRD compared with females of low area SES (RR 1.20, CI 1.002–1.459, *p* = .043).

### Ethnic disparities in type 1 diabetes mellitus and type 2 diabetes mellitus treated end stage renal disease in aboriginal and Torres Strait islanders, Maori and Pacific islanders

The number of Aboriginal and Torres Strait Islanders and Maori and Pacific Islanders with ESRD from T1DM was very low, whilst the RR for all other Australians and New Zealanders in comparison to Aboriginal and Torres Strait Islanders, Maori and Pacific Islanders was 2.99 (CI 2.14–4.18, *p* < 0.001) [30]. For ESRD in the setting of T2DM however the RR was 6.57 for Indigenous Australians (CI 6.04–7.14, *p* < 0.001) and 6.48 for Maori and Pacific Islanders (CI 6.02–6.97, *p* < 0.001) [30] in comparison to all other Australians and New Zealanders.

## Discussion

### Population prevalence of diabetes

The prevalence of diabetes in the Australian population follows a pronounced inverse social gradient, increasing incrementally as area SES falls. It was previously reported that the national prevalence of diabetes was 5.4% and that low area SES was associated with a threefold higher prevalence of T2DM [6], but the striking and uniform social gradient demonstrated in this study has not been described previously. A recent study in Germany has also demonstrated a social gradient in the prevalence of diabetes [31] suggesting that this may be a global phenomenon. Across the Australian population there was also a statistically significant gradient in T1DM with the highest incidence also seen in the lowest area SES. Although the incidence of T1DM shows a large variation worldwide, environmental or genetic causation has not yet been determined [32] and this is the first Australian study to describe an inverse relationship between incidence of T1DM and area SES.

### Type 1 diabetes mellitus and treated end stage renal disease

The mean age of presentation with T1DM ESRD (45 years) is significantly lower than for other aetiologies combined (other than paediatric-specific kidney disease) with the national mean age of presentation with ESRD being 60 years [33]. There were few T1DM ESRD cases diagnosed after the age of 60 years, probably because of higher mortality seen in T1DM [34]. There was an excess of ESRD for males with T1DM in Australia and this may be due to several factors. There is an overall higher incidence of T1DM in males [7], higher cardiac mortality has been demonstrated for females with T1DM [35, 36] resulting in death before the development of ESRD, a higher risk for males of developing ESRD regardless of disease aetiology [33] and a bias in propensity to treat towards males [37]. This study has also shown an increased risk of ESRD for males of low area SES. There is considerable evidence for poorer outcomes in people of low SES with T1DM [10, 14] and of the higher risk of ESRD for males [38, 39], however no other studies to date have found an increased risk of ESRD only for males of low area SES. A recent Swedish study found that being of low SES increased the risk of death two- or three-fold, and that males were more likely to die overall, but this was not explicitly examined in relation to males of low SES [40]. Health care access is an important social determinant of health outcomes in T1DM and Australia offers a Universal ‘free’ health care service. However people with low SES have lower levels of health literacy defined as more than literacy and numeracy to incorporate a degree of difficulty in navigating the health care service irrespective of ‘universal coverage’ [41]. Australian research is warranted to explore the higher risk of ESRD for males of low area SES to determine if it is an adverse outcome or in fact a survival advantage.

### Aboriginal and Torres Strait islanders, Maori and Pacific islanders

The very low incidence of T1DM ESRD in the Aboriginal and Torres Strait Islander and Maori populations, in the face of the extremely high incidence of T2DM ESRD, is difficult to interpret for a number of reasons. The issue of survival may be an underlying factor because there is a well-demonstrated intergenerational cyclical disadvantage experienced by Indigenous people as a result of colonisation [42]. Aboriginal and Torres Strait Islander people have the worst health, lowest life expectancy and highest child mortality rates of all Australians [43] and death rates due to diabetes, which is generally reported as T2DM in this population, are 30 times higher than in other Australians [44]. In addition, there appears to be no studies of young adult onset T1DM in the Aboriginal and Torres Strait Islander population despite Australian data demonstrating that almost 50% of cases of T1DM are diagnosed after the age of 15 years [1, 8]. Studies conducted overseas suggest that up to 30% of cases diagnosed as T2DM test positive for autoantibodies and could be due to T1DM or late onset autoimmune diabetes in adults (LADA) [45]. This raises a second possibility, that of misclassification and conflation of T1DM with T2DM, which could lead to incorrect treatment and earlier death. These findings in relation to T1DM suggest a need to investigate diabetes classification in the Aboriginal and Torres Strait Islander population with autoantibody and C-peptide testing. It is also worth noting that in regard to the grossly inflated risk of ESRD due to T2DM in Aboriginal and Torres Strait Islanders, Maori and Pacific Islanders, these populations also have the lowest SES, and being an ethnic minority and of low SES are independently associated with poor outcomes in diabetes [46].

### Study limitations

Reporting of census data to the Australian Bureau of Statistics who develop the SEIFA categories is mandatory in Australia. The risk of ecological fallacy is acknowledged when determining individual SES status from area SES through the assumptions that are made about individuals based on group data [47]. However, the purpose of this study is not to determine causation but to make large scale comparisons between groups [22]. The use of SEIFA categories has repeatedly demonstrated that health status is related to socioeconomic status however cannot identify exactly which aspects of socioeconomic disadvantage lead to health disadvantage [48]. In addition there may be status inconsistencies in the SEIFA SES areas. This study reports a sub-sample of the Australian population for diabetes prevalence in relation to SES and a whole of population study would be needed to be fully conclusive. The association between area SES and ESRD could be a consequence of people moving to an area of low SES because of chronic illness and reduced capacity for income. In addition, there are competing risks for ESRD development in relation to the high cardiac mortality seen in T1DM. A diagnosis bias to T2DM for a case of T1DM cannot be excluded when using registry data and there may be inconsistent classification of ethnicity for Aboriginal and Torres Strait Islanders, Maori and Pacific Islanders. Data relating to prevalence and renal outcomes of T1DM in the Aboriginal and Torres Strait Islander, Maori and Pacific Islander population is thought to be widely underreported and insufficient for conclusions to be drawn. The 5 year time frame used in this study is a limitation and may have impacted on the strength of the results. The data in Table 1 describing the incidence of T1DM ESRD would be more fully understood with the addition of standardized rates/reference population however to our knowledge no such data on T1DM is kept in Australia due to the absence of a T1DM registry.

## Conclusion

This study has demonstrated a uniform inverse social gradient in diabetes prevalence in Australia confirming the strong association between socioeconomic disadvantage and the disease. With regard to diabetic nephropathy ESRD whilst it has previously been demonstrated using ANZDATA that populations in Australia of low area SES carry a heavier burden of ESRD this study of T1DM ESRD over a five-year period shows that there appears to be an increased risk only for males of low area SES. ESRD may not be the most suitable measure of disproportionate outcomes in T1DM in Australia given that a high mortality from the disease for people of low SES before reaching ESRD is likely. Other countries have reported an inverse relationship between SES and mortality in T1DM [10–14] with a much higher likelihood of death for people who are socioeconomically disadvantaged and this disparity is more pronounced in T1DM than in T2DM [49]. For this reason, it remains uncertain whether this study demonstrates an increased risk for males or a survival advantage. While there are doubts about classification of diabetes in the Aboriginal and Torres Strait Islander population, the grossly inflated risk of diabetic nephropathy ESRD for Aboriginal and Torres Strait Islanders, Maori and Pacific Islanders over the time period studied is a shared disparate outcome across these three minority populations that has changed little in the past 20 years.

## Abbreviations

ANZDATA: Australia and New Zealand Dialysis and Transplant Registry
ESRD: End Stage Renal Disease
RR: Relative Risk
SEIFA: Socioeconomic Indexed for Areas
SES: Socioeconomic Status
T1DM: Type 1 Diabetes Mellitus
T2DM: Type 2 Diabetes Mellitus

## Footnotes

1 Data are presented in box and whisker plots with outliers (extreme cases in each decile) represented by the symbols °* which denote individual postcodes.

2 The postcode with zero prevalence seen in decile 1 (lowest area SES) in Fig. 2 is a remote area and may represent lack of services and/or registration with the National Diabetes Services Scheme.
